# Developmentally adapted cognitive processing therapy for adolescents and young adults with PTSD symptoms after physical and sexual abuse: study protocol for a randomized controlled trial

**DOI:** 10.1186/1745-6215-15-195

**Published:** 2014-05-29

**Authors:** Rita Rosner, Hans-Helmut König, Frank Neuner, Ulrike Schmidt, Regina Steil

**Affiliations:** 1 Department of Clinical and Biological Psychology, Catholic University Eichstätt-Ingolstadt, Ostenstr. 25, Eichstätt 85071, Germany; 2 Department of Health Economics and Health Services Research, University Medical Center Hamburg-Eppendorf, Martinistr. 52, Hamburg 20246, Germany; 3 Department of Clinical Psychology and Psychotherapy, Bielefeld University, Bielefeld 33501, Germany; 4 Max-Planck-Institute of Psychiatry, Kraepelinstr. 2-10, Munich 80804, Germany; 5 Department of Clinical Psychology and Psychotherapy, Institute of Psychology, Goethe University Frankfurt, Varrentrappstr. 40-42, Frankfurt am Main 60054, Germany

**Keywords:** Abuse, Adolescents, Cognitive processing therapy, Post-traumatic stress disorder

## Abstract

**Background:**

Although childhood sexual and/or physical abuse (CSA/CPA) is known to have severe psychopathological consequences, there is little evidence on psychotherapeutic interventions for adolescents and young adults suffering from post-traumatic stress disorder (PTSD). Equally sparse are data on moderators of treatment response on PTSD-related epigenetic changes, health care costs and loss of productivity, alterations in cognitive processing, and on how successful interventions affect all of these factors. Early treatment may prevent later (co)morbidity. In this paper, we present a study protocol for the evaluation of a newly developed psychotherapeutic manual for PTSD after CSA/CPA in adolescents and young adults – the Developmentally Adapted Cognitive Processing Therapy (D-CPT).

**Methods/design:**

In a multicenter randomized controlled trial (RCT) D-CPT is compared to treatment as usual (TAU). A sample of 90 adolescent outpatients aged 14 to 21 years will be randomized to one of these conditions. Four assessments will be carried out at baseline, at end of treatment, and 3 and 6 months after end of therapy. Each time, patients will be assessed via clinical interviews and a wide range of questionnaires. In addition to PTSD symptoms and comorbidities, we will evaluate moderators of treatment response, epigenetic profiles, direct and indirect costs of this disorder, and neurophysiological processing of threat cues in PTSD and their respective changes in the course of these two treatments (D-CPT and TAU).

**Discussion:**

The study will provide new insights in the understudied field of PTSD in adolescents and young adults. A newly developed intervention will be evaluated in this therapeutically underserved population. Results will provide data on treatment efficacy, direct and indirect treatment costs, as well as on associations of treatment outcome and PTSD intensity both to epigenetic profiles and to the neurobiological processing of threat cues. Besides, they will help to learn more about the psychopathology and possible new objective correlates of PTSD.

**Trial registration:**

Germanctr.de identifier: DRKS00004787.

## Background

Unfortunately, traumatic experiences in childhood and adolescence are common: a meta-analysis based on 65 international studies indicates that nearly 20% of women and 8% of men experienced sexual abuse prior to the age of 18 [1]. Regarding physical abuse in Western and European nations, numbers range from 3.6% to 16.3% [2-5]. These negative childhood experiences do not only have short-term consequences concerning the victims’ mental health – they are also related to a number of psychiatric disorders during the whole life span [6]. Consequently, people exposed to childhood sexual abuse (CSA) have a 2.4 heightened risk for the development of psychopathology compared to those without such experiences. For childhood physical abuse (CPA), this factor is around 1.5 [7]. In a meta-analysis, mortality and morbidity, drug and alcohol misuse, risky sexual behavior, obesity, and criminal behavior were found as consequences of childhood maltreatment [8]. According to Cutajar et al. [9], CSA increases the risk for several mental diseases like psychosis, affective, anxiety, substance abuse, and personality disorders. There is a particularly high probability for the development of post-traumatic stress disorders (PTSD) – exposure to CSA leads to an increased PTSD risk of 5.6 compared to non-CSA exposure. Accordingly, international studies show a high prevalence for PTSD after CSA, which ranges from 37% to 44% [10,11]. However, victims of CSA do not only suffer from PTSD; comorbidity secondary to PTSD often develops during adolescence and early adulthood, moderated by the patient’s efforts to fight painful trauma-related emotions, for instance by using harmful substances and by developing suicidal, self-injurious, or other severely dysfunctional behavior [12,13]. Early pregnancies and re-victimization occur at higher odds than in the general population [14,15].

Given these negative consequences, it is obvious that abuse-related PTSD should be treated at an early stage. For children, there already exist evaluated interventions: a meta-analysis of 39 studies analyzing treatment results for children and adolescents with psychiatric symptoms after CSA reported a general effect size of *g* = 0.77 for PTSD symptoms in the 5 studies with controlled designs and an untreated comparison control group. For the other studies, the authors report pre-post effect sizes of *g* = 1.13 for PTSD [16]. In contrast, few randomized controlled trials (RCTs) for PTSD therapy of adolescents were included in this meta-analysis – only two studies enrolling adolescents over 14 years of age used control conditions (waiting list/supportive treatment) and randomization [17,18]. However, as the mean age in both studies was around 11 years, the majority of participants were children. To our knowledge, there exist only five studies examining treatment effects on post-traumatic stress symptoms after CPA or CSA among adolescent patients exclusively [19-23]. Being the first of their kind, these studies are undoubtedly important. However, most of them have methodological deficits like caseness not defined by PTSD diagnoses or the use of insufficient diagnostic instruments. Only the study of Foa et al. [23] meets the standards for controlled studies, although it only focusses on girls. In summary, adolescents beyond the age of 14 are inadequately represented in PTSD research so far.

Thus, the major goal of the presented project herein is to provide information on treatment efficacy in this understudied group. A highly effective cognitive behavioral therapy manual for adults, Cognitive Processing Therapy (CPT [24]), was adapted for adolescent patients with CSA/CPA-related PTSD [25] – the Developmentally Adapted Cognitive Processing Therapy (D-CPT). Four major adjustments were integrated in the protocol. First of all, the treatment intensity was increased by administering the middle part of the protocol in high frequency (approximately 15 sessions during four weeks) to enhance the adolescents’ motivation; applying treatment in such intensity was reported to be superior to the usual scheme of one session per week [26]. Second, a commitment phase was included in the protocol in order to further build treatment motivation and to enhance therapeutic alliance as well as to establish the formal framework that is needed to conduct therapy. As survivors of childhood abuse often experience difficulties in emotion regulation, behavior and emotion management techniques, as used within Dialectical Behavior Therapy for PTSD (DBT-PTSD [27,28]), were also integrated in D-CPT. Finally, special consideration was given to developmental tasks affecting the patient’s entire life, such as career choice, vocational training, and romantic relationships. Adolescent patients are at high risk of school or secondary education drop out, of starting relationships with abusive partners, or of becoming re-victimized. Therefore, we decided to specifically address these issues at the end of treatment.

D-CPT has already been successfully piloted; nearly all of the 12 participating patients showed large reductions of post-traumatic stress symptoms and, moreover, comorbid symptoms improved significantly as well [25]. In October 2012, our research group started its work on the RCT and the associated studies. This article introduces its study protocol.

### The current trial

The overall aim of this RCT is to demonstrate the efficacy of a newly adapted psychotherapy intervention protocol in comparison to treatment as usual (TAU) in the understudied population of physically and sexually abused adolescents and young adults suffering from PTSD. Severity of PTSD symptoms before and after therapy serves as a primary outcome. Secondary goals are to evaluate changes in general psychopathology and comorbidity, such as depression, borderline personality features, and dissociation. Thirdly, the trial will allow for an estimation of the efficacy of TAU in Germany, on which currently no data is available.

Adjunct projects will provide new insights in questions beyond treatment efficacy; electroencephalographic (EEG) parameters are collected in an experiment which aims to find neuronal correlates of threat processing evoked by different categories of emotional words. Preliminary studies could show that anxiety disorders modify the way threatening information is processed [29]. By adapting these studies to the current project, multiple research questions may be answered. Namely, it will be possible to investigate differences in information processing between healthy subjects (control group), traumatized but healthy subjects (trauma control group), and traumatized subjects with PTSD (experimental group). In addition to this, the influence of D-CPT on information processing can be assessed on a psychophysiological level.

On the basis of questionnaires and videotaped sessions, potential predictors of treatment success are analyzed. As there is little research about handling PTSD in adolescents, also important variables influencing therapy outcome, such as treatment adherence, therapist competence, and working alliance, are not well studied [30]. Equally sparse is data about societal costs for the treatment of PTSD and/or mental comorbidities like depression or anxiety disorders (direct costs) as well as PTSD-related loss of productivity (indirect costs). No study has ever investigated the indirect costs resulting from PTSD in adolescents and, so far, no study has analyzed any cost data of PTSD or PTSD-treatment in Germany. Using different questionnaires, these issues are addressed in the D-CPT-project.

Concerning the genetic basis of PTSD and therapy outcome, there are only very few (though promising) clinical studies indicating epigenetic changes in PTSD in humans (for example [31]). In our study, the analysis of epigenetic profiles of PTSD patients by saliva samples during the course of therapy is realized via high-throughput epigenetic profiling.

## Methods/design

### Trial design

D-CPT is an open, rater-blinded, multicenter, 2-arms RCT comparing D-CPT to TAU in Germany. There are five assessments: at baseline, within the treatment (approx. 8 weeks after beginning), directly after end of treatment (post-treatment) as well as 3 and 6 months after end of treatment (follow-up 1 and 2).

### Study centers

**Figure 1:**
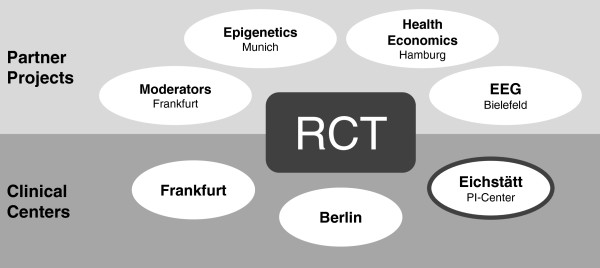
**The D-CPT network and its study centers.** RCT, Randomized controlled trial; PI, Principal investigator.

The D-CPT project is designed as a multicenter study with Rita Rosner (University Eichstätt-Ingolstadt) as principal investigator. Treatment is offered in three university outpatient clinics: Freie Universität Berlin (supervised by Babette Renneberg), Goethe University Frankfurt am Main (supervised by Regina Steil), and University Eichstätt-Ingolstadt (supervised by Rita Rosner). Moreover, there are four partner projects at different research sites. Besides being a clinical center, the work group at Goethe University Frankfurt am Main (Regina Steil) studies potential predictors of treatment outcome. The analysis of epigenetic profiles of PTSD patients by saliva samples during the course of therapy is the task of a research group from the Max-Planck Institute of Psychiatry in Munich (Ulrike Schmidt). At the University Medical Center Hamburg-Eppendorf (Hans-Helmut König), direct and indirect costs of the disorder are evaluated. Bielefeld University (Frank Neuner) examines the neurophysiological processing of threat cues in PTSD and their respective changes in the course of the treatments. The D-CPT network and its study centers are displayed in Figure 1.

### Participants

Adolescents and young adults aged 14 to 21 years may take part in the study. For inclusion, PTSD as a primary diagnosis after experiencing CSA and/or CPA beyond the age of three, according to the definition of the American Psychological Association [32], is required. To be diagnosed with PTSD, patients must meet 1 B-criterion, 2 C-criteria, and 2 D-criteria in the Clinician Administered PTSD Scale for Children and Adolescents (CAPS-CA [33]). A stable psychopharmacological medication is necessary at study entry, i.e., in the last 3 weeks there was either no or a constant psychopharmacological medication. Moreover, living in safe conditions, a sufficient knowledge of German language, as well as an informed consent from the parents/legal guardians (if under 18) and the participant is compulsory.

Exclusion criteria are:

• Acute suicidality within the last 6 months

• Life threatening self-harming behavior within the last 6 months

• Substance-related or organic mental disorder

• Pervasive developmental disorder

• Acute or lifetime diagnosis of a psychotic disorder according to the Diagnostic and Statistical Manual of Mental Disorders, Fourth Edition (DSM-IV-TR [34])

• Acute or lifetime diagnosis of a bipolar disorder according to DSM-IV-TR

• Current diagnosis of substance dependence according to DSM-IV-TR (abstinence <6 months)

• Mental retardation (IQ ≤75)

• Simultaneous psychological or psychiatric treatment

### Planned interventions

Patients are enrolled either to TAU or D-CPT. Patients in TAU will be advised to look for treatment with other therapists in the respective areas. A detailed instruction to find a psychotherapist via internet will be given to the client by the coordinator of the respective study center. TAU patients will be monitored at the corresponding measurement points planned for D-CPT. After follow-up 1, patients can be enrolled into D-CPT if they wish to do so.

D-CPT is organized into four phases [25]. The treatment begins with a commitment phase comprising 5 sessions in 4 weeks. The aim of this phase is to build a therapeutic alliance and to increase and test the adolescents’ motivation for treatment. Safety plans are developed and anti-suicide contracts are signed. In phase 2, patients learn to tolerate and control intensive trauma-related emotions without acting in a dysfunctional way. For this reason, an emotion regulation training based on DBT-PTSD [27] is applied with 6 sessions in 4 weeks. Phase 3 comprises 15 sessions of CPT, according to Resick et al. [24], administered in high frequency (within 4 weeks); this is the central part of D-CPT. Dysfunctional beliefs related to the traumatic experiences are identified and modified by cognitive restructuring. In the last phase of treatment, developmental tasks specific to adolescence are introduced in 4 sessions during 4 weeks (one weekly session). The goal of this final therapy phase is to minimize the risk of future victimization and/or to prevent the choice of abusive partners. Moreover, the last treatment sessions focus on supporting the patient to complete education and to get an adequate vocational training. In sum, D-CPT comprises 30 sessions with 6 optional sessions for crisis intervention or communication with care givers or other institutions. Table 1 summarizes the treatment design and content. Adherence to D-CPT will be ensured by manualization, continuous local as well as central supervision, and ratings of therapeutic adherence and competence of random samples of videotaped sessions. Therapists are trained within one 4-day and one 2-day workshop. After attending the first workshop, every therapist treats at least one pilot case.

**Table 1:** Overview of D-CPT

**Week**	**Sessions**	**Phase**	**Details**
1–4	5	Commitment	Therapy contract, emergency plan
			Lifeline
			Therapy goals
5–8	6	Emotion-regulation	Monitoring and identifying dysfunctional behavior and its long-term consequences
			Using distress tolerance skills
			Education about emotions
9–12	15	Intensive CPT	PTSD symptoms and psychological processes
			Identifying maladaptive beliefs (“stuck points”)
			Remembering the traumatic event through written accounts
			Challenging stuck points
			Focusing on special themes including safety, trust, control, esteem, and intimacy
13–16	4	Developmental tasks	Education about potentially abusive partners
			Education-focused help
			Inclusion of social network
			Review

### Outcome measurements

#### ***Primary outcome***

Severity of PTSD is measured at each assessment point using the CAPS-CA [33] (in German [35]). The CAPS-CA is a structured clinical interview which rates frequency and intensity of PTSD symptoms on a scale ranging from 0 (never) to 4 (daily or almost daily) and from 0 (none) to 4 (extreme), respectively. According to the German interview guidelines [35], a symptom is recorded as present if both frequency and intensity are at least rated with 1.

### Secondary outcome

A self-rating of PTSD symptoms is obtained weekly within therapy and at every assessment point using the University of California Los Angeles PTSD Reaction Index (UCLA [36], in German [37]).

At pre- and post-treatment as well as at follow-up 1 and 2 assessments, psychiatric comorbidity is measured with the Structured Clinical Interview for DSM Disorders (SCID [38,39], in German [40]), further important parts of the Diagnostic Interview for Mental Disorders in Childhood and Adolescence (Kinder-DIPS [41]), and the nicotine section of the Expert System for Diagnosing Mental Disorders (DIA-X [42]).

Moreover, patients are asked to complete a wide range of questionnaires measuring comorbidity and related problems at every assessment before and after treatment. Among these are:

• Trauma Symptom Checklist for Children (TSCC [43], in German [44])

• Beck Depression Inventory (BDI-II [45], in German [46])

• Adolescent Dissociative Experiences Scale (A-DES [47], in German [48])

• Borderline Symptom List 23 (BSL-23 [49])

• Youth Self-Report (YSR [50], in German [51])

• Modified version of the Client Sociodemographic and Service Receipt Inventory (CSSRI [52], in German [53])

• EuroQol 5 Dimensions (EQ-5D [54], in German [55])

Besides, saliva samples are collected for high-throughput epigenetic profiling and psychophysiological parameters are assessed by EEG – each at baseline and follow-up 1.

### Additional measures

Participants are tested with the Culture-Fair Intelligence Test (CFT-20-R [56]), which ensures that patients meet the cognitive requirements for treatment.

Detailed information about abuse are obtained by the Childhood Trauma Questionnaire (CTQ [57], in German [58]) and the Interview for Traumatic Events in Childhood (ITEC [59]). The ITEC is a retrospective, semi-structured interview for childhood maltreatment that provides dimensional scores for the severity of sexual, physical, and emotional abuse as well as emotional and physical neglect.

For the examination of mediators and moderators, like therapeutic alliance, maladaptive beliefs, and mental images, the Helping Alliance Questionnaire [60] (in German [61]), the Post-traumatic Maladaptive Beliefs Scale for Youth (PMBSY; Matulis S, Resick PA, Steil R, personal communication, 2012), and an instrument for the measurement of mental images (FVB; Schreiber F, Gutermann J, Matulis S, Steil R, personal communication, 2012) are used. Emotion regulation is assessed using the Affective Style Questionnaire (ASQ [62], in German [63]) and attachment is studied by the Experiences in Close Relationships – Revised Questionnaire (ECR-R [64], in German [65]). Therapeutic adherence and competence are rated on the basis of randomly drawn videographies of treatment sessions by independent raters using two newly developed rating scales, the D-CPT Adherence Scale [66] and the Cognitive Therapy Competence Scale for D-CPT [66].

### Procedure

**Figure 2:**
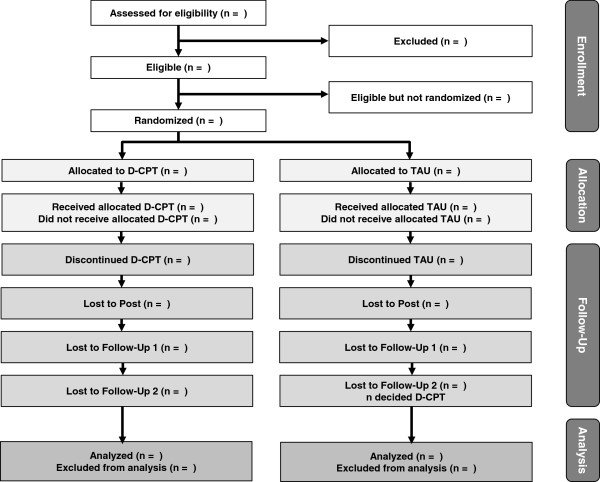
**CONSORT flow diagram.** D-CPT, Developmentally adapted cognitive processing therapy; TAU, Treatment as usual.

Patients will be sent by private practitioners, hospitals, school psychologists, and children’ services as well as child and adolescent mental health services. Homepages, newspaper reports, and leaflets in schools will support individual referrals. In a first interview, patients are screened for inclusion and exclusion criteria. If suitable, patients are informed about study conditions and asked to give informed consent. When this requirement is met, pre-diagnostic procedures comprising SCID, Kinder-DIPS, DIA-X, CAPS-CA, ITEC, CFT-20-R, several questionnaires (YSR, CSSRI, EQ-5D, ECR-R, ASQ, BDI-II, A-DES, BSL-23, CTQ, UCLA, TSCC, PMBSY, FVB), saliva sampling, and an EEG session are carried out. Patients are randomized to either TAU or D-CPT subsequently. After emotion-regulation training, patients in D-CPT are invited to complete process measures including the CAPS-CA and UCLA. For patients in TAU, this measurement is arranged around 8 weeks after randomization. After the end of treatment, post-diagnosis (directly after treatment), follow-up 1 (3 months after end of treatment), and follow-up 2 (6 months after end of treatment) measurements take place. These involve the SCID, Kinder-DIPS, DIA-X, CAPS-CA, and several questionnaires (YSR, CSSRI, EQ-5D, ECR-R, ASQ, BDI-II, A-DES, BSL-23, UCLA, TSCC, PMBSY, FVB) at each assessment point, as well as saliva collection and EEG at follow-up 1. These assessments are conducted by independent and trained interviewers who are blind to the treatment condition. Figure 2 shows the flow diagram of participant recruitment according to CONSORT [67].

### Sample size calculation

A type I error of 0.05 and a statistical power of 0.8 were used for sample size calculations. As there exists no effect size estimation for TAU in Germany, we suggest an effect size of d = 0.3. Based on the data reported by Harvey and Taylor [16], an effect size of d = 0.9 was assumed for D-CPT. Thus, two groups of n = 45 patients are required for the trial. No adjustment for drop-out is necessary as last observation carried forward substitution of the major study endpoint will be performed during the intention-to-treat analysis.

### Statistical analysis

To test the hypothesis that D-CPT is superior to TAU, a confirmatory analysis of the study will be calculated as a *t*-test of post-treatment scores of the CAPS-CA. The critical value of alpha will be set to 0.05. Additional analyses focusing on secondary study endpoints or on generating hypotheses for subsequent studies will be performed on an exploratory basis using up-to-date sophisticated statistical models.

### Safety and ethical issues

The trial has been approved by the Ethics Committee of Catholic University Eichstätt-Ingolstadt and is conducted according to the ICH Guideline for Good Clinical Practice (ICH-GCP [68]). Prior to assessments, all patients are informed about study design and procedures including randomization, probable side effects, and the possibility of ending the participation at any time without disadvantages. Informed consent has to be provided by participating patients and by their parents or guardian if the adolescent is younger than 18 years of age. For safety reasons, suicidal tendencies are constantly observed during assessment and treatment. If necessary, patients will be referred to cooperating clinics. Serious adverse events (SAEs) are continuously monitored and reported through the whole trial. In correspondence with the ICH-GCP, the following incidents are defined as SAEs:

• A physical disease with the risk of permanent impairment and a following medical intervention

• Inpatient somatic hospitalization with a minimum of one overnight stay or prolongation of existing hospitalization

• Inpatient psychiatric hospitalization with a minimum of one overnight stay or prolongation of existing hospitalization (including emergency accommodation/crisis intervention service)

• Intoxication with psychotropic substances (including alcohol) and following medical intervention

• Delinquency with judiciary or police contact

• External aggression to property (statutory offense)

• External aggression to persons (statutory offense)

• Intentional self-harming behavior requiring medical aid

• Attempted suicide

• Re-victimization including all traumatic events according to DSM-IV-TR

In case of a SAE, the responsible therapist or study coordinator reports the event to his/her supervisor and an independent Data and Safety Monitoring Board (DSMB). The decision, whether there is a causal link between treatment and occurrence of SAEs, is taken by the DSMB. If severe safety concerns become apparent to the DSMB, the trial will be stopped.

If an investigator has ethical concerns because of the performance at one of the centers, the coordinating investigator or the co-principal investigator will be informed immediately. The coordinating investigator and the co-principal investigator are authorized to discontinue interventions in a trial center because of insufficient and/or inadequate recruitment, insufficient quality of data, or specific problems unforeseeable in advance which render the continuation of the study impossible at that specific center.

## Discussion

Although the severe psychopathological consequences of sexual and/or physical abuse are known, only few studies report on the results of trauma focused psychotherapy for adolescents [19-23]. Consequently, chances were hitherto missed to administer effective treatments in early life to prevent the development of severe comorbidity or re-victimization. Reasons for the lack of studies are manifold, and might be due to difficulties in psychological assessments differing for adolescents and young adults, as well as to legal issues around different licensing procedures for therapists treating children and adolescents and those treating adult patients. Moreover, the lack of studies might be related to the characteristics of adolescents, whose motivational instability, rapid and intensive mood changes, and disentanglement from family structures have the potential to impede treatment participation and completion. The only published study on a similar methodological level needed 6 years of randomization to finally include 61 adolescent girls [23]. This highlights once more the specific problems in this age group. To bridge this knowledge gap by introducing a developmentally adapted CPT is the goal of the D-CPT network. The novelty of D-CPT is the adaption of the CPT procedure to the group of traumatized adolescents by increasing treatment intensity, integrating emotion-regulation-training, and finally by considering developmental tasks. The overall aim of the project is to evaluate this new therapy. Besides, the network aims to address several other heavily underexplored issues like variables influencing treatment outcome, epigenetics, health economics, and neurophysiological processing.

Taken together, the innovative character of our project is represented in i) a study sample of adolescents and young adults suffering from PTSD, ii) the developmentally adapted version of CPT (D-CPT) whose feasibility for youth within the German health system will be evaluated, and iii) measurement of the so far unknown effectiveness of TAU in Germany, which will be estimated.

Moreover, issues addressed in the D-CPT partner projects have a pioneering value. Predictors of treatment success like treatment adherence, therapist competence, and working alliance are not as well studied in PTSD as in other disorder specific interventions [30]. The same applies to epigenetics and PTSD; to our knowledge, the D-CPT study will be the first clinical study analyzing epigenetic profiles of PTSD patients during the course of therapy [69] and it will be the first survey screening for epigenetic markers for PTSD in adolescents and young adults. Likewise, research on health economic evidence is rare for PSTD. Last but not least, the novelty in the examination of neurophysiological processing of threat cues in PTSD lies in the differentiation between maltreated subjects with and without psychopathology.

In summary, the D-CPT project will provide insight in a wide range of different issues concerning PTSD and its treatment in adolescents and young adults. We are confident that, in the long term, it will contribute to the improvement of psychotherapeutic care for this therapeutically underserved population.

The project is part of a comprehensive network about behavioral disorders related to violence, neglect, maltreatment, and abuse in childhood and adolescence.

## Trial status

The trial started in October 2012 and will end in 2015/2016. Patient enrolment is ongoing with 31 of the 90 participants having been recruited.

## Abbreviations

BDI-II: Beck Depression Inventory; ASQ: Affective Style Questionnaire; CAPS-CA: Clinician Administered PTSD Scale for Children and Adolescents; CPA: Childhood physical abuse; CPT: Cognitive Processing Therapy; CSA: Childhood sexual abuse; CSSRI: Client Sociodemographic and Service Receipt Inventory; DBT: Dialectical Behavior Therapy; D-CPT: Developmentally Adapted Cognitive Processing Therapy; DIA-X: Expert System for Diagnosing Mental Disorders; DSMB: Data and Safety Monitoring Board; DSM-IV-TR: Diagnostic and Statistical Manual of Mental Disorders, Fourth Edition; ECR-R: Experiences in Close Relationships – Revised; EEG: Electroencephalogram; EQ-5D: EuroQol 5 Dimensions; FVB: Fragebogen zur Erfassung mentaler Vorstellungsbilder; ICH-GCP: ICH Guideline for Good Clinical Practice; Kinder-DIPS: Diagnostic Interview for Assessment of Psychological Disorders in Childhood and Adolescence; PI: Principal Investigator; PMBSY: Post-traumatic Maladaptive Beliefs Scale for Youth; PTSD: Post-traumatic stress disorder; RCT: Randomized controlled trial; SAE: Serious adverse event; SCID: Structured Clinical Interview for DSM Disorders; TAU: Treatment as usual; UCLA: University of California Los Angeles PTSD Reaction Index; YSR: Youth Self-Report.

## Competing interests

The authors declare that they have no competing interests.

## Authors’ contributions

RR is the Principal Investigator on the D-CPT trial. RR and RS did the major work on the design of the RCT and oversee data collection at their clinical center. HHK, FN, US, and RS are Project Coordinators on the trial and participated in the design of the study. All authors read and approved the final manuscript.

## References

[B1] Pereda N Guilera G Forns M Gomez-Benito J The prevalence of child sexual abuse in community and student samples: a meta-analysis Clin Psychol Rev 2009 29 328 338 10.1016/j.cpr.2009.02.007 19371992

[B2] Annerback EM Sahlqvist L Svedin CG Wingren G Gustafsson PA Child physical abuse and concurrence of other types of child abuse in Sweden-Associations with health and risk behaviors Child Abuse Negl 2012 36 585 595 10.1016/j.chiabu.2012.05.006 22854707

[B3] Elklit A Victimization and PTSD in a Danish national youth probability sample J Am Acad Child Adolesc Psychiatry 2002 41 174 181 10.1097/00004583-200202000-00011 11837407

[B4] Finkelhor D Turner H Ormrod R Hamby SL Violence, abuse, and crime exposure in a national sample of children and youth Pediatrics 2009 124 1411 1423 10.1542/peds.2009-0467 19805459

[B5] Hawkins AO Danielson CK de Arellano MA Hanson RF Ruggiero KJ Smith DW Saunders BE Kilpatrick DG Ethnic/racial differences in the prevalence of injurious spanking and other child physical abuse in a national survey of adolescents Child Maltreat 2010 15 242 249 10.1177/1077559510367938 20498129

[B6] Stirling J Jr Amaya-Jackson L Understanding the behavioral and emotional consequences of child abuse Pediatrics 2008 122 667 673 18762538 10.1542/peds.2008-1885

[B7] Fergusson DM Boden JM Horwood LJ Exposure to childhood sexual and physical abuse and adjustment in early adulthood Child Abuse Negl 2008 32 607 619 10.1016/j.chiabu.2006.12.018 18565580

[B8] Gilbert R Widom CS Browne K Fergusson D Webb E Janson S Child maltreatment 1. Burden and consequences of child maltreatment in high-income countries Lancet 2009 373 68 81 10.1016/S0140-6736(08)61706-7 19056114

[B9] Cutajar MC Mullen PE Ogloff JR Thomas SD Wells DL Spataro J Psychopathology in a large cohort of sexually abused children followed up to 43 years Child Abuse Negl 2010 34 813 822 10.1016/j.chiabu.2010.04.004 20888636

[B10] McLeer SV Deblinger E Henry D Orvaschel H Sexually abused children at high risk for post-traumatic stress disorder J Am Acad Child Adolesc Psychiatry 1992 31 875 879 10.1097/00004583-199209000-00015 1400120

[B11] McLeer SV Dixon JF Henry D Ruggiero K Escovitz K Niedda T Scholle R Psychopathology in non-clinically referred sexually abused children J Am Acad Child Adolesc Psychiatry 1998 37 1326 1333 10.1097/00004583-199812000-00017 9847506

[B12] Giaconia RM Reinherz HZ Silverman AB Pakiz B Frost AK Cohen E Traumas and posttraumatic stress disorder in a community population of older adolescents J Am Acad Child Adolesc Psychiatry 1995 34 1369 1380 10.1097/00004583-199510000-00023 7592275

[B13] Essau CA Conradt J Petermann F Häufigkeit der Posttraumatischen Belastungsstörung bei Jugendlichen: Ergebnisse der Bremer Jugendstudie Z Kinder Jugendpsychiatr Psychother 1999 27 37 45 10.1024//1422-4917.27.1.37 10096158

[B14] Barnes JE Noll JG Putnam FW Trickett PK Sexual and physical revictimization among victims of severe childhood sexual abuse Child Abuse Negl 2009 33 412 420 10.1016/j.chiabu.2008.09.013 19596434 PMC2723796

[B15] Noll JG Shenk CE Putnam KT Childhood sexual abuse and adolescent pregnancy: a meta-analytic update J Pediatr Psychol 2009 34 366 378 10.1093/jpepsy/jsn098 18794188 PMC2722133

[B16] Harvey ST Taylor JE A meta-analysis of the effects of psychotherapy with sexually abused children and adolescents Clin Psychol Rev 2010 30 517 535 10.1016/j.cpr.2010.03.006 20417003

[B17] King NJ Tonge BJ Mullen P Myerson N Heyne D Rollings S Martin R Ollendick TH Treating sexually abused children with posttraumatic stress symptoms: a randomized clinical trial J Am Acad Child Adolesc Psychiatry 2000 39 1347 1355 10.1097/00004583-200011000-00008 11068889

[B18] Cohen JA Mannarino AP Knudsen K Treating sexually abused children: 1 year follow-up of a randomized controlled trial Child Abuse Negl 2005 29 135 145 10.1016/j.chiabu.2004.12.005 15734179

[B19] Sinclair JJ Larzelere RE Paine M Jones P Graham K Jones M Outcome of group treatment for sexually abused adolescent females living in a group home setting: preliminary findings J Interpers Violence 1995 10 533 542 10.1177/088626095010004011

[B20] Danielson CK McCart MR Walsh K de Arellano MA White D Resnick HS Reducing substance use risk and mental health problems among sexually assaulted adolescents: A pilot randomized controlled trial J Fam Psychol 2012 26 628 635 22686269 10.1037/a0028862 PMC3419329

[B21] Tourigny M Hébert M Daigneault I Simoneau AC Efficacy of a group therapy for sexually abused adolescent girls J Child Sex Abus 2005 14 71 93 10.1300/J070v14n04_04 16354649

[B22] Krakow B Sandoval D Schrader R Keuhne B McBride L Yau CL Tandberg D Treatment of chronic nightmares in adjudicated adolescent girls in a residential facility J Adolesc Health 2001 29 94 100 10.1016/S1054-139X(00)00195-6 11472867

[B23] Foa EB McLean CP Capaldi S Rosenfield D Prolonged exposure vs supportive counseling for sexual abuse-related PTSD in adolescent girls: a randomized clinical trial JAMA 2013 310 2650 2657 10.1001/jama.2013.282829 24368465

[B24] Resick PA Monson CM Chard KM Cognitive Processing Therapy: Veteran/Military Version 2008 Washington, DC: Department of Veterans’ Affairs

[B25] Matulis S Resick PA Rosner R Steil R Developmentally adapted cognitive processing therapy for adolescents suffering from posttraumatic stress disorder after childhood sexual or physical abuse: a pilot study Clin Child Fam Psychol Rev 2014 17 2 173 190 10.1007/s10567-013-0156-9 24101403

[B26] Ehlers A Clark DM Hackmann A Grey N Liness S Wild J Manley J Waddington L McManus F Intensive cognitive therapy for PTSD: a feasibility study Behav Cogn Psychother 2010 38 383 398 10.1017/S1352465810000214 20573292 PMC2893530

[B27] Steil R Dyer A Priebe K Kleindienst N Bohus M Dialectical behavior therapy for posttraumatic stress disorder related to childhood sexual abuse: a pilot study of an intensive residential treatment program J Trauma Stress 2011 24 102 106 10.1002/jts.20617 21351167

[B28] Bohus M Dyer AS Priebe K Krüger A Kleindienst N Schmahl C Niedtfeld I Steil R Dialectical behaviour therapy for post-traumatic stress disorder after childhood sexual abuse in patients with and without borderline personality disorder: a randomised controlled trial Psychother Psychosom 2013 82 221 233 10.1159/000348451 23712109

[B29] Wabnitz P Martens U Neuner F Cortical reactions to verbal abuse: event-related brain potentials reflecting the processing of socially threatening words Neuroreport 2012 23 774 779 10.1097/WNR.0b013e328356f7a6 22797317

[B30] Barber JP Triffleman E Marmar C Considerations in treatment integrity: Implications and recommendations for PTSD research J Trauma Stress 2007 20 793 805 10.1002/jts.20295 17955529

[B31] Uddin M Aiello AE Wildman DE Koenen KC Pawelec G DeLos Santos R Goldmann E Galea S Epigenetic and immune function profiles associated with posttraumatic stress disorder Proc Natl Acad Sci U S A 2010 107 9470 9475 10.1073/pnas.0910794107 20439746 PMC2889041

[B32] American Psychological Association Guidelines for psychological evaluations in child protection matters Am Psychol 2013 68 20 31 23025746 10.1037/a0029891

[B33] Nader KO Kriegler JA Blake DD Pynoos RS Clinical Administered PTSD Scale, Child and Adolescent Version (CAPS-C) 1994 White River Junction, VT: National Center for PTSD

[B34] APA (American Psychiatric Assosication) Dt Bearb V, Sass H, Wittchen HU, Zaudig M, Houben I Diagnostisches und Statistisches Manual Psychischer Störungen - Textrevision - DSM-IV-TR 2003 Göttingen: Hogrefe

[B35] Steil R Füchsel G IBS-KJ. Interviews zu Belastungsstörungen bei Kindern und Jugendlichen. Diagnostik der akuten und der posttraumatischen Belastungsstörung 2006 Göttingen: Hogrefe

[B36] Steinberg AM Brymer MJ Decker KB Pynoos RS The University of California at Los Angeles post-traumatic stress disorder reaction index Curr Psychiatry Rep 2004 6 96 100 10.1007/s11920-004-0048-2 15038911

[B37] Ruf M Schauer M Elbert T Barkmann C, Schulte Markwort M, Brähler E UPID – UCLA PTSD Index for DSM IV Fragebögen zur Diagnostik psychischer Störungen des Kindes- und Jugendalters 2010 Göttingen: Hogrefe

[B38] First MB Spitzer R Gibbon M Williams J Benjamin L Structured Clinical Interview for DSM-IV Axis II Personality Disorders (SCID II) 1994 New York: Biometric Research Department

[B39] First MB Spitzer RL Gibbon M Williams JBW Structured Clinical Interview for DSM-IV Axis I disorders (SCID I) 1997 New York: Biometric Research Department

[B40] Wittchen H-U Zaudig M Frydrich T Strukturiertes Klinisches Interview für DSM-IV. Achse I und II 1997 Göttingen: Hogrefe

[B41] Schneider S Unnewehr S Margraf J Diagnostisches Interview bei psychischen Störungen im Kindes- und Jugendalter (Kinder-DIPS) 2009 2 Berlin: Springer 10.1024/1422-4917//a000247 23988834

[B42] Wittchen HU Pfister H DIA-X-Interviews: Manual für Screening Verfahren und Interview; Interviewheft Längsschnittuntersuchung (DIA-X-Lifetime); Ergänzungsheft (DIA-X-Lifetime); Interviewheft Querschnittsuntersuchung (DIA-X-12 Monate); Ergänzungsheft (DIA-X-12 Monate); PC-Programm zur Durchführung des Interviews (Längs- und Querschnittsuntersuchung); Auswertungsprogramm 1997 Frankfurt: Swets & Zeitlinger

[B43] Briere J Trauma Symptom Checklist for Children (TSCC) Professional Manual 1996 Odessa, FL: Psychological Assessment Resources

[B44] Matulis S Loos L Steil R Validierung der deutschen adaptation der trauma symptom checklist for children [abstract] Trauma & Gewalt. Abstractband 2014 146

[B45] Beck AT Steer RA Brown GK Manual for the Beck Depression Inventory-II 1996 San Antonio, TX: The Psychological Corporation

[B46] Hautzinger M Keller F Kühner C BDI-II. Beck Depressions-Inventar Revision 2006 Frankfurt: Harcourt Test Services

[B47] Armstrong JG Putnam FW Carlson EB Libero DZ Smith SR Development and validation of a measure of adolescent dissociation: the adolescent dissociative experiences scale J Nerv Ment Dis 1997 185 491 497 10.1097/00005053-199708000-00003 9284862

[B48] Brunner R Resch F Parzer P Koch E Heidelberger Dissoziations-Inventar (HDI): Manual 2008 Frankfurt/Main: Pearson

[B49] Bohus M Kleindienst N Limberger MF Stieglitz R-D Domsalla M Chapman AL Steil R Philipsen A Wolf M The short version of the Borderline Symptom List (BSL-23): development and initial data on psychometric properties Psychopathology 2009 42 32 39 10.1159/000173701 19023232

[B50] Achenbach TM Manual for the Youth Self-Report and 1991 Profile 1991 Burlington: University of Vermont, Department of Psychiatry

[B51] Döpfner M Fragebogen für Jugendliche; deutsche Bearbeitung der Youth Self Report Form der Child Behavior Checklist (YSR) 1998 Köln: Arbeitsgruppe Kinder-, Jugend- und Familiendiagnostik

[B52] Chisholm D Knapp MRJ Knudsen HC Amaddeo F Gaite L van Wijngaarden B Client socio-demographic and service receipt inventory – European version: development of an instrument for international research: EPSILON Study 5 Br J Psychiatry Suppl 2000 177 s28 s33 10.1192/bjp.177.39.s28 10945075

[B53] Roick C Kilian R Matschinger H Bernert S Mory C Angermeyer MC German adaptation of the client sociodemographic and service receipt inventory - an instrument for the cost of mental health care Psychiatr Prax 2001 28 84 90 11605129 10.1055/s-2001-17790

[B54] Group EQ EuroQol – a new facility for the measurement of health-related quality of life Health policy 1990 16 3 199 208 10109801 10.1016/0168-8510(90)90421-9

[B55] von der Schulenburg JMG Claes C Greiner W Uber A Die deutsche Version des EuroQol-Fragebogens Z Gesundh Wiss 1998 6 3 20 10.1007/BF02956350

[B56] Weiß RH Grundintelligenztest Skala 2 – Revision CFT 20-R 2006 Göttingen: Hogrefe

[B57] Bernstein DP Fink L Childhood Trauma Questionnaire. A retrospective self-report 1998 Orlando: The Psychological Corporation

[B58] Bader K Hänny C Schäfer V Neuckel A Kuhl C Childhood Trauma Questionnaire - Psychometrische Eigenschaften einer deutschsprachigen Version Z Klin Psychol Psychother 2009 38 223 230 10.1026/1616-3443.38.4.223

[B59] Lobbestael J Arntz A Harkema-Schouten P Bernstein D Development and psychometric evaluation of a new assessment method for childhood maltreatment experiences: the Interview for Traumatic Events in Childhood (ITEC) Child Abuse Negl 2009 33 505 517 10.1016/j.chiabu.2009.03.002 19758701

[B60] Alexander LB Luborsky L Greenberg LS, Pinsof WM The Penn Helping Alliance Scales The Psychotherapeutic Process: A Research Handbook. Guilford Clinical Psychology and Psychotherapy Series 1986 New York, NY: Guilford Press 325 366

[B61] Bassler M Potratz B Krauthauser H Der “Helping Alliance Questionnaire” (HAQ) von Luborsky: Möglichkeiten zur Evaluation des therapeutischen Prozesses von stationärer Psychotherapie Psychotherapeut 1995 40 23 32

[B62] Hofmann SG Kashdan TB The affective style questionnaire: development and psychometric properties J Psychopathol Behav Assess 2010 32 255 263 10.1007/s10862-009-9142-4 20495674 PMC2873215

[B63] Graser J Bohn C Kelava A Schreiber F Hofmann SG Stangier U Der “Affective Style Questionnaire” (ASQ): Deutsche Adaption und Validitäten Diagnostica 2012 58 100 111 10.1026/0012-1924/a000056

[B64] Fraley RC Waller NG Brennan KA An item response theory analysis of self-report measures of adult attachment J Pers Soc Psychol 2000 78 350 365 10707340 10.1037//0022-3514.78.2.350

[B65] Ehrenthal JC Dinger U Lamla A Funken B Schauenburg H Evaluation der deutschsprachigen Version des Bindungsfragebogens “Experiences in Close Relationships – Revised” (ECR-RD) Psychother Psychosom Med Psychol 2009 59 215 223 10.1055/s-2008-1067425 18600614

[B66] Gutermann J Schreiber F Matulis S Steil R Reliabilität der Skalen zur Erfassung psychotherapeutischer Adhärenz und Kompetenz in der Behandlung von Jugendlichen mit Posttraumatischer Belastungsstörung (PTBS) nach Gewalterfahrungen [abstract] Trauma & Gewalt. Abstractband 2014 145

[B67] Schulz KF Altman DG Moher D CONSORT 2010 statement: updated guidelines for reporting parallel group randomized trials Ann Intern Med 2010 152 726 732 10.7326/0003-4819-152-11-201006010-00232 20335313

[B68] ICH Harmonised Tripartite Guideline Guideline for Good Clinical Practice E6 http://www.ich.org/fileadmin/Public_Web_Site/ICH_Products/Guidelines/Efficacy/E6_R1/Step4/E6_R1__Guideline.pdf 11832605

[B69] Schmidt U Kaltwasser SF Wotjak CT Biomarkers in posttraumatic stress disorder: overview and implications for future research Dis Markers 2013 35 12 10.1155/2013/835876 PMC3774961 24167348

